# Prophylactic Allogeneic Hematopoietic Stem Cell Therapy for *CSF1R* ‐Related Leukoencephalopathy

**DOI:** 10.1002/mds.29011

**Published:** 2022-05-19

**Authors:** Morten Andreas Horn, Anders Eivind Myhre, Trine Prescott, Jan Aasly, Christina Heidemann Sundal, Tobias Gedde‐Dahl

**Affiliations:** ^1^ Department of Neurology Oslo University Hospital Oslo Norway; ^2^ Department of Hematology Oslo University Hospital Oslo Norway; ^3^ Section of Medical Genetics, Department of Laboratory Medicine Telemark Hospital Trust Skien Norway; ^4^ Department of Neurology St. Olavs Hospital Trondheim Norway; ^5^ Department of Neuroscience NTNU Trondheim Norway; ^6^ Department of Neurology NeuroClinic Lillestrøm Norway; ^7^ Department of Neurology Sahlgrenska Academy, University of Gothenburg Gothenburg Sweden; ^8^ Division of Medicine Institute for Clinical Medicine, University of Oslo Oslo Norway

Recently, Tipton and coworkers^1^ reported on the results of allogeneic hematopoietic stem cell therapy (HSCT) in 7 individuals with *CSF1R*‐related leukoencephalopathy. HSCT appeared to slow progression in most of them after about 6 months. An unresolved question is whether presymptomatic HSCT prevents the development of leukoencephalopathy in patients with pathogenic *CSF1R* variants or ameliorates the course if leukoencephalopathy subsequently develops.

We report the results of prophylactic HSCT in a 31‐year‐old woman who is heterozygous for the pathogenic *CSF1R* variant NM_005211.3(CSF1R): c.1754‐2A>G previously detected in her family. She is the daughter of NO‐2 in the report by Rademakers et al^2^ (Fig 1) and was clinically and radiologically unaffected at presentation. All affected relatives had rapidly devastating disease, resulting in death within 3 years of diagnosis.

**FIG 1:**
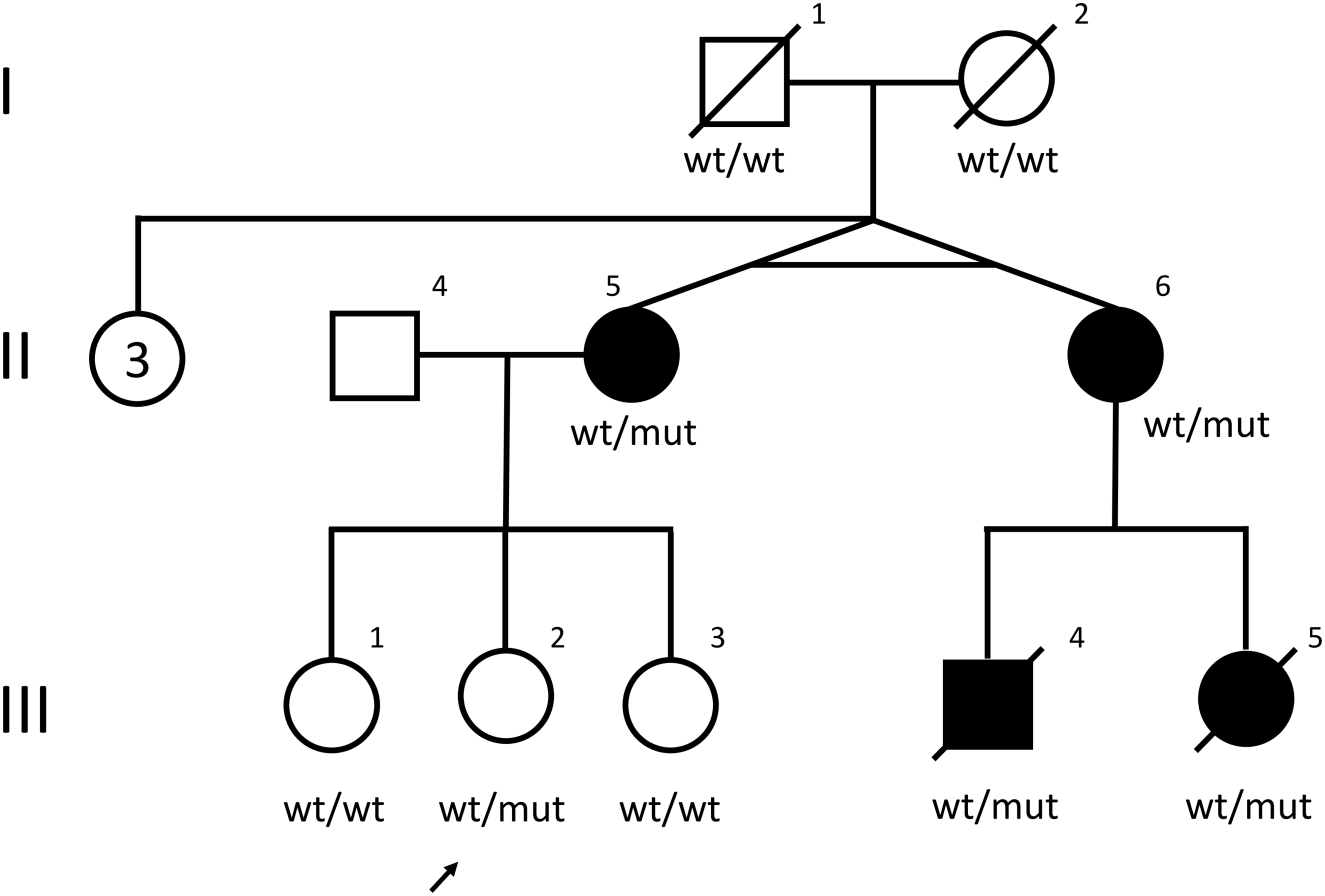
Pedigree of the Norwegian family with *CSF1R*‐related leukoencephalopathy. Symbols: 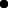 = affected female, 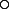 = unaffected female, 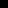 = affected male, 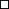 = unaffected male, / = deceased individual, wt/wt = homozygous for wild‐type (normal) *CSF1R* allele, wt/mut = heterozygous for pathogenic *CSF1R* variant. Arrow points to individual III:2, recipient of prophylactic HSCT. Monozygotic twins II:5 and II:6 are NO‐2 and NO‐1, respectively, in Rademakers et al.^2^ Cause of death in all affected individuals was *CSF1R*‐related leukoencephalopathy: II:5 died at age 40 years, II:6 at age 41 years, III:4 at age 36 years, and III:5 at age 31 years.

Given the uniformly fatal outcome in her relatives, the woman requested HSCT, based on previous case reports.^3^, ^4^, ^5^ HSCT was discussed at length with her and her family. The decision to provide treatment followed multiple rounds of discussion among neurologists, hematologists, and geneticists nationally, as well as consultation with expertise from France and the United States. HSCT was performed in August 2020, using the Mayo Clinic reduced intensity conditioning regimen^1^ with a 9/10 HLA‐matched unrelated male donor bone marrow graft. Post‐transplantation she received cyclophosphamide, sirolimus, and mycophenolate as graft versus host disease (GvHD) prophylaxis without pretreatment to open the blood–brain barrier. Engraftment was observed at day +20, and complete donor chimerism reached at day +27. Lumbar puncture at +12 months showed 99% donor chimerism in the cerebrospinal fluid; T‐cells, B‐cells, and monocytes were defined by morphology and flow cytometry. During the first 12 months post‐transplantation, there were no manifestations of GvHD.

Clinical neurologic testing was unremarkable before HSCT and at +12 months. Cerebral magnetic resonance imaging (MRI) at −28, −11, −4, −3, and 0 months before HSCT was normal and remained so at +4, +8, and +12 months. Detailed neuropsychological testing performed at −5 months and repeated at +12 months was unremarkable.

One year after transplantation, the woman became subacutely ill. Her MRI was compatible with inflammation of the left temporal lobe, brainstem, medulla, and meninges. Mild pleocytosis was present in the cerebrospinal fluid, but no infectious agents were detected, and there was no evidence of malignancy or autoimmune disease despite extensive investigations. An allogeneic immunological reaction seems a probable but unproven cause. Clinically, she was mildly affected with subtle signs of myelopathy and modest cognitive difficulties. She improved spontaneously without medical intervention.

To our knowledge, this is the first report of allogeneic HSCT performed with the aim of preventing progressive, potentially fatal *CSF1R*‐related leukoencephalopathy in an asymptomatic individual. At +17 months, evidence of leukoencephalopathy is absent. This is encouraging but is, of course, not proof of efficacy. The decision to provide HSCT was ethically challenging. We remain uncertain as to whether asymptomatic individuals with pathogenic *CSF1R* variants should be provided HSCT. Bridging therapies that could stem the progression of leukoencephalopathy,^6^ so that HSCT is delayed until MRI changes develop, might be a better option.

## Author Roles

1. Research project: A. Conception, B. Organization, C. Execution;
2. Statistical analysis: A. Design, B. Execution, C. Review and critique;
3. Manuscript: A. Writing of the first draft, B. Review and critique.

M.A.H.: 1A, 1B, 1C, 3A, 3B

C.H.S.: 1A, 3B

A.E.M.: 1A, 1B, 1C, 3B

T.P.: 1B, 3B

J.A.: 1A, 3B

T.G.‐D.: 1A, 1B, 1C, 3B

## Full financial disclosures for the previous 12 months

M.A.H.: none.

C.H.S.: advisory board for Novartis, Teva, and Allergan.

A.E.M.: none.

T.P.: none.

J.A.: none.

T.G.‐D.: advisory board for Novartis, Incyte, and Takeda.

## Data Availability

Data sharing is not applicable to this article as no new data were created or analyzed in this study.
